# Notch signaling activation contributes to cardioprotection provided by ischemic preconditioning and postconditioning

**DOI:** 10.1186/1479-5876-11-251

**Published:** 2013-10-08

**Authors:** Xue-liang Zhou, Li Wan, Qi-rong Xu, Yong Zhao, Ji-chun Liu

**Affiliations:** 1 Department of Cardiac Surgery, The First Affiliated Hospital, Nanchang University, Nanchang 330006, China

**Keywords:** Notch signaling, Mitochondrial permeability transition pore, Stat3, Ischemic preconditioning, Ischemic postconditioning

## Abstract

**Background:**

Notch signaling is known to be activated following myocardial ischemia, but its role in cardioprotection provided by ischemic preconditioning (IPC) and ischemic postconditioning (IPost) remains unclear.

**Methods:**

Lentiviral vectors were constructed to overexpress or knockdown N1ICD in H9c2 cardiomyocyte and rat heart exposed to ischemia reperfusion injury (IRI), IPC or IPost.

**Results:**

Notch1 signaling was activated during myocardial IPC and IPost, and could enhance cell viability and inhibit apoptosis. Furthermore, activated Notch1 signaling stabilized mitochondrial membrane potential and reduced reactive oxygen species induced by IRI. The cardioprotection provided by activated Notch1 signaling resembled that of IPC and IPost, which was related to Stat3 activation and regulation of apoptosis related proteins. Furthermore, in langendorff heart perfusion model, activated Notch1 signaling restored cardiac function, decreased lactate dehydrogenase release and limited infarct size after myocardial ischemia. Conclusions: Notch1 signaling is activated and mediates cardioprotection provided by IPC and Ipost. Notch1 signaling may represent a potential new pharmacologic mimic for cardioprotection of ischemic heart disease.

## Introduction

It is well known that immediate restoration of cardiac blood flow after prolonged ischemia would contradictorily result in ischemia reperfusion injury (IRI), which would accelerate the deterioration of cardiac function [1]. Ischemia preconditioning (IPC) has shown beneficial effects such as limiting infarct size and reducing lethal arrhythmia after IRI in many species including human being [2,3]. On the other hand, ischemia postconditioning (IPost) indicates brief episodes of nonlethal ischemia/reperfusion applied at the onset of reperfusion [4]. Accumulating evidence indicates that IPC and IPost activate various survival signaling pathways to provide cardioprotection [5,6]. Therefore, developing pharmacologic mimics such as endogenous autacoids to provide myocardial protection have important clinical significance for the treatment of ischemic heart disease (IHD).

Notch signaling is an evolutionary conserved signaling pathway which plays a crucial role in cell fate decision, cell differentiation, proliferation, and apoptosis [7]. There are four receptors (Notch1-4) and five ligands (Jagged1-2 and Delta like 1, -3,-4) in mammals, the hairy and enhancer of split (Hes) and hes-related (Hey) families of transcriptional repressors have been identified as Notch classical target genes [8]. Notch signaling has been implicated in heart disease. Notch1 and Jagged1 levels were lower in adult heart, but N1ICD, Jagged1 and Delta4 were accumulated in the border zone of the infarct region at 4 days after ischemia [9]. Quantitative RT-PCR analysis revealed that the expression of Notch1-4, Jagged1 and Hes1 were increased within the first week following myocardial infarct [10]. Notch signaling was activated in cardiomyocytes during post-infarction remodeling, and the expression of Notch1, Jagged1 and Hes1 were upregulated in dilated or hypertrophic hearts [11]. In addition, Notch signaling can stimulate immature cardiomyocyte proliferation and promote quiescent cardiomyocytes to reenter the cell cycle [12,13]. However, whether activated Notch signaling following myocardial ischemia plays cardioprotective effects during IPC and IPost remains unclear.

Therefore, in this study we investigated the role of Notch1 signaling in cardioprotection provided by IPC and IPost, using both cardiomyocyte and rat heart model. Our results showed that the inhibition of Notch1 signaling via the knockdown of N1ICD abrogated the cardioprotection provided by IPC and IPost.

## Materials and methods

### Reagents and antibodies

Dulbecco’s modified Eagle’s medium (DMEM) and trypsin were from Gibco BRL (USA). Fetal bovine serum (FBS) was from NQBB International Biological Corporation (Australia). Cell counting kit-8 (CCK-8) and Reactive oxygen species assay kits were from Beyotime institute of biotechnology (China). Annexin V-FITC apoptosis detection kit was obtained from KeyGEN Biotech(China). Mitochondria staining kit (JC-1) was purchased from MultiSciences Biotech (USA). Mitochondria isolation kit was purchased from Applygen Technologies (China). LDH kits were purchased from Nanjing jiancheng bioengineering institute, 2,3,5-triphenyltetrazolium chloride (TTC) was from SolarbioTechnologies (China). Enhanced chemiluminescence kit was purchased from Thermo Scientific (USA).

Notch1 and Hes1 antibodies were purchased from Abcam (USA), Flag, Bcl-xL and Bax antibodies were purchased from Cell Signaling Technology (USA), Stat3, p-Stat3 and β-Actin antibodies were purchased from Anbo Biotechnology Company (USA).

### Cell culture

Rat cardiac H9c2 cells (ATCC Rockville, USA) were cultured in DMEM supplemented with 10% FBS at 37°C in a humidified incubator with 5% CO_2_. The cells were fed every 3 days and subcultured once reaching 90% confluence. Cells were plated at an appropriate density according to each experimental design.

### Virus vector construction and infection

Rat N1ICD cDNA (Notch1:5456–7819) was generated by PCR using forward primer: 5′-GAGGATCCCCGGGTACCGGTCGCCACCATGGTGCTGCTGTCCCGCAAG-3′ and reverse primer: 5′-TCATCCTTGTAGTCGCTAGCCTTAAATGCCTCTGGAATG-3′. The amplified fragment was subcloned into pGC-FU-EGFP-3Flag to get pGC-FU-N1ICD-3Flag vector. Rat N1ICD-shRNAs were designed according to Ambion with the following sequences: GCACAGTGCTGAGTACCAA (N1ICD-shRNA-1), GCCTCTCCACCAATACCTT (N1ICD-shRNA-2), CCCACATTCCAGAGGCATT (N1ICD-shRNA-3), TCTGGATGCCCGAATGCAT (N1ICD-shRNA-4) with CTCGAG as the loop sequence. The oligonucleotide-annealed products were subcloned into pGVC112 to get pGVC112-N1ICD-shRNA vectors. An irrelevant TTCTCCGAACGTGTCACGT was used as negative control.

To generate lentiviral vectors, either 20 μg pGC-FU-N1ICD-3Flag or pGVC112-N1ICD-shRNA was transfected along with 15 μg pHelper 1.0 and 10 μg pHelper 2.0 into 293T cells in 10 cm plate at 80% confluence for 8 h. The medium was replaced with fully supplemented DMEM and the supernatants were harvested after 72 h for virus titration, which were then stored at −80°C.

To infect H9c2 cells, multiplicity infection (MOI) of 20 lentiviral particles with 5 μg/ml polybrene were added into the medium, 24 h later the medium was changed and H9c2 cells were cultured for an additional 72 h.

To infect Sprague–Dawley rat, 100 μl pseudo viral particles (1×10^8^/ml) were injected into the tail vein, 4 weeks after infection, Sprague–Dawley rats were collected for analysis.

### In vitro protocols of hypoxia/reoxygenation (H/R), IPC, IPost

For H/R, H9c2 cells were cultured in hypoxic solution in a hypoxic incubator (95% N_2_-5% CO_2_) for 3 h, subsequently hypoxic solution was replaced with reoxygenation solution, and the cells were cultured in a high oxygen incubator (95% O_2_-5% CO_2_) for 3 h.

For IPC, H9c2 cells were cultured in hypoxic solution in a hypoxic incubator (95% N_2_-5% CO_2_) for 10 min, subsequently the hypoxic solution was replaced with reoxygenation solution, and the cells were cultured in a high oxygen incubator (95% O_2_-5% CO_2_) for 10 min. The hypoxia-reoxygenation cycle was repeated 3 times and followed by H/R treatment.

For IPost, at the end of the 3 h hypoxia, the hypoxic solution was replaced with reoxygenation solution and H9c2 cells were cultured in a high oxygen incubator for 10 min. Next the medium was changed to hypoxic solution and the cells were cultured in a hypoxic incubator for another 10 min. The reoxygenation-hypoxia cycle was repeated 3 times and followed by continuous culture in reoxygenation solution in a high oxygen incubator for 3 h.

### Cell viability assay

H9c2 cells were seeded into a 96-well plate at 10,000 cells/well and cultured in 100 μL DMEM supplemented with 10% FBS for 24 h, then treated as indicated. Finally, the cell viability was detected using CCK-8 Kit (Dojindo, Japan) according to the manufacturer’s instructions. The absorbance was measured at 450 nm using a Microplate reader (Thermo, USA). The percentage of viable cells was calculated with the control cells set as 100%.

### Apoptosis assay

H9c2 cells were harvested and 4×10^5^ cells were resuspended in 500 μl binding buffer, then incubated with 5 μl Annexin V-FITC and 5 μl propidium iodide (PI) at room temperature in dark for 15 min. The samples were analyzed by flow cytometry with a BD FACSCalibur. Apoptosis ratio was defined as the ratio between Annexin V positive/PI negative cells and the total living cells.

### Detection of intracellular reactive oxygen species (ROS)

H9c2 cells were harvested and 10^5^ cells were incubated with 10 μmol/L DCFH-DA at 37°C for 20 min. The DCF fluorescence was detected by BD FACSCalibur excited at 488 nm and the emitted fluorescence was collected at 525 nm.

### Determination of mitochondrial membrane potential (ΔΨm)

H9c2 cells were harvested and 10^5^ cells were loaded with JC-1 (2 μM) at 37°C, 5% CO_2_ for 20 min, then analyzed by BD FACSCalibur excited at 488 nm and the emitted fluorescence was collected at 530 nm and 590 nm. ΔΨm was expressed as the emitted fluorescence ratio (590/530 nm) in percentage to the initial level, whereas 0% level was recorded after complete mitochondrial depolarization with carbonyl cyanide-p-trifluoromethoxyphenylhydra-zone.

### Langendorff heart perfusion

Male Sprague–Dawley rats (weight 220 to 250 g) were obtained from Jiangxi University of Traditional Chinese Medicine (China). All animals were treated in accordance with the Guide for the Care and Use of Laboratory Animals, and approved by the Ethics Committee of the first affiliated hospital of Nanchang University (permit No: NDYFY-2012-YYLSD-016). The rats were anesthetized with pentobarbital sodium (100 mg/kg intraperitoneally). Hearts were excised and retrograde perfused via the aorta with modified Krebs-Henseleit buffer (NaCl 118 nM, KCl 4.7 nM, MgSO_4_ 1.2 nM, CaCl_2_ 2.5 nM, NaHCO_3_ 2 nM, KH_2_PO_4_ 1.2 nM, Glucose 11 nM, pH 7.35-7.45) equilibrated with 95% O_2_-5% CO_2_. Left ventriculardeveloped pressure (LVDP), heart rate (HR) and intraventricular pressure (±dP/dt) were measured by Powerlab pressure transducer with a balloon inserted into the left ventricle. In the Control, Mock1 and Mock2 groups, hearts were buffer perfused without ischemia. In ischemia/reperfusion (I/R) group, hearts were first equilibrated for 20 min followed by 30 min of stop-flow ischemia and 120 min of reperfusion. In IPC group, after 3 cycles of 5 min of ischemia and 5 min of reperfusion, hearts were subjected to 30 min of no-flow ischemia, followed by 120 min of reperfusion. In IPost group, hearts were subjected to 30 min of stop-flow ischemia, before 120 min of reperfusion, 3 cycles of 10 s of ischemia and 10 s of reperfusion were applied.

### Lactate dehydrogenase (LDH) assay

Coronary effluent was collected at 1 min before ischemia, 1 min after ischemia and 1 min after reperfusion to determine LDH activity by using LDH kits according to the manufacturer’s instructions. LDH activity was expressed as international units per liter (IU/L).

### Assessment of infarct size

The heart was frozen at −20°C for 30 min, then sectioned from apex to base into five to six 1 mm sections. The sections were incubated in 1%TTC at 37°C for 30 min and fixed in 10% formaldehyde for 24 h. The normal myocardium was stained brick red with TTC while the infarcted portion was not stained and appeared pale white. The infarct area-IA (white) and the area at risk-AAR (red and white) from each section were measured using Image Pro 6.0 Plus software. For each section, infarct size was expressed as the percentage of IA/AAR.

### Western blot analysis

At different time points of the experiemtns, cells or the hearts were harvested immediately and lysed in cell lysis buffer (Beyotime Institute of Biotechnology, China) at 4°C. Protein samples were separated by 8-10% SDS-PAGE, then transferred to nitrocellulose membranes (Millipore) and blocked in 10% nonfat milk in TBST (150 mM NaCl, 50 mM Tris pH 7.5, 0.1% Tween-20). Membranes were incubated with primary antibodies overnight at 4°C, followed by incubation with secondary antibodies at room temperature for 1 h. The fluorescent signals were detected using enhanced chemiluminescence by ImageQuant LAS4000 (GE, USA). Relative protein levels were normalized to that of β-actin.

### Statistical analysis

Data were analyzed using the SPSS version 12 statistical analysis package (SPSS Inc., Chicago, IL, USA). Examined data were assessed using ANOVA. In each test, the data were expressed as the mean±SD, and P<0.05 was accepted as statistically significant.

## Results

### Identification of LV-N1ICD and LV-N1ICD-shRNA vectors

**Figure 1:**
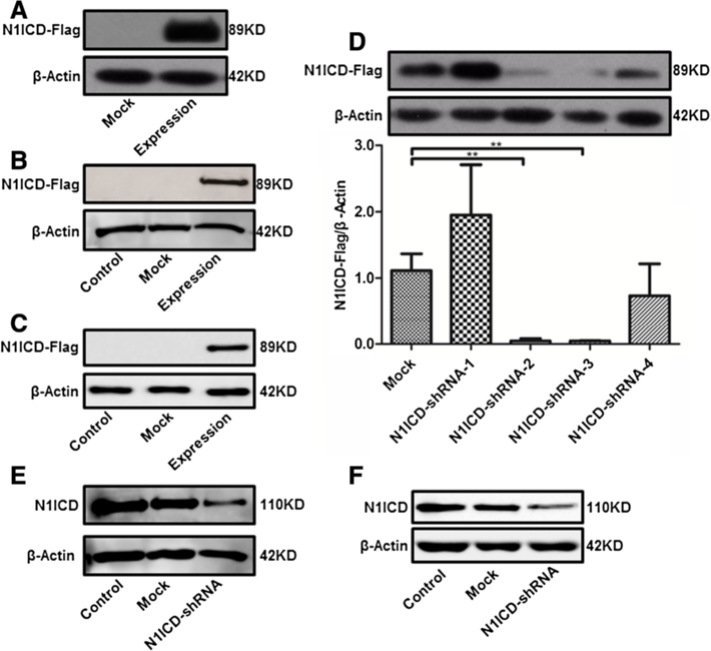
**Identification of LV-N1ICD and LV-N1ICD-shRNA vectors. A**, Western blots showing the expression of Flag tagged N1ICD in 293T cells as indicated. **B**, Western blots showing the expression of Flag tagged N1ICD in H9c2 cells as indicated. **C**, Western blots showing the expression of Flag tagged N1ICD in the hearts of the rats as indicated, **D**, Western blots showing the expression of Flag tagged N1ICD in 293T cells transfected with pGC-FU-N1ICD-3Flag along with negative control pGVC112-shRNA (Mock), pGVC112-N1ICD-shRNA-1 (N1ICD-shRNA-1), pGVC112-N1ICD-shRNA-2 (N1ICD-shRNA-2), pGVC112-N1ICD-shRNA-3 (N1ICD-shRNA-3), or pGVC112-N1ICD-shRNA-4 (N1ICD-shRNA-4). **E**, Western blots showing the expression of endogenous N1ICD in H9c2 cells infected by lentivirus as indicated, **F**, Western blots showing the expression of endogenous N1ICD in the hearts of the rats infected by lentivirus as indicated. β-actin was loading control.

In 293T cells transfected with pGC-FU-N1ICD-3Flag vector, we observed ectopic expression of N1ICD (Figure 1A). Furthermore, in H9c2 cells and rats infected with lentiviral vector LV-N1ICD, we observed ectopic expression of N1ICD in the cells or hearts (Figure 1B-C). In contrast, in 293T cells cotransfected with pGC-FU-N1ICD-3Flag vector and N1ICD-shRNA-1 to 4, we observed significant knockdown of N1ICD expression in cells transfected with N1ICD-shRNA-3 (Figure 1D). Thus we constructed N1ICD lentiviral interference vector (LV-N1ICD-shRNA) using the sequence of N1ICD-shRNA-3 and infected H9c2 cells or rats, we observed efficient knockdown of endogenous N1ICD expression in the cells or hearts (Figure 1E-F). Taken together, these results suggest that LV-N1ICD and LV-N1ICD-shRNA vectors are efficient to overexpress and knockdown N1ICD in vitro and in vivo, respectively.

### Notch1 signaling is activated during myocardial IPC and IPost

**Figure 2:**
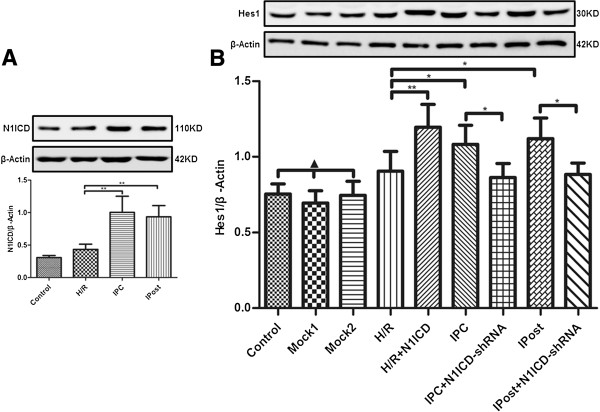
**Notch1 signaling is activated in H9c2 cells exposed to IPC and IPost. A**, N1ICD level was upregulated in IPC and IPost. **B**, Hes1 level was regulated by H/R, IPC and IPost with or without LV-N1ICD/LV-N1ICD-shRNA infection. Data were presented as mean±SD, n=3. Mock1 indicated cells infected with empty vector, Mock2 indicated cells infected with vector containing nonspecfic sequences. ▲*P*>0.05, *P<0.05, **P<0.01.

Western blot analysis showed that N1ICD and Hes1 expression in H9c2 cells was increased slightly during H/R but increased significantly during IPC and IPost (Figure 2A and 2B). In addition, Hes1 level was increased in H9c2 cells treated by LV-N1ICD infection and H/R, but decreased in H9c2 cells treated by LV-N1ICD-shRNA infection and IPC or IPost (Figure 2B), confirming that Hes1 is a target gene of Notch signaling. These data suggest that Notch1 signaling is activated during myocardial IPC and IPost.

### Myocardial IRI is mitigated by activated Notch1 signaling

**Figure 3:**
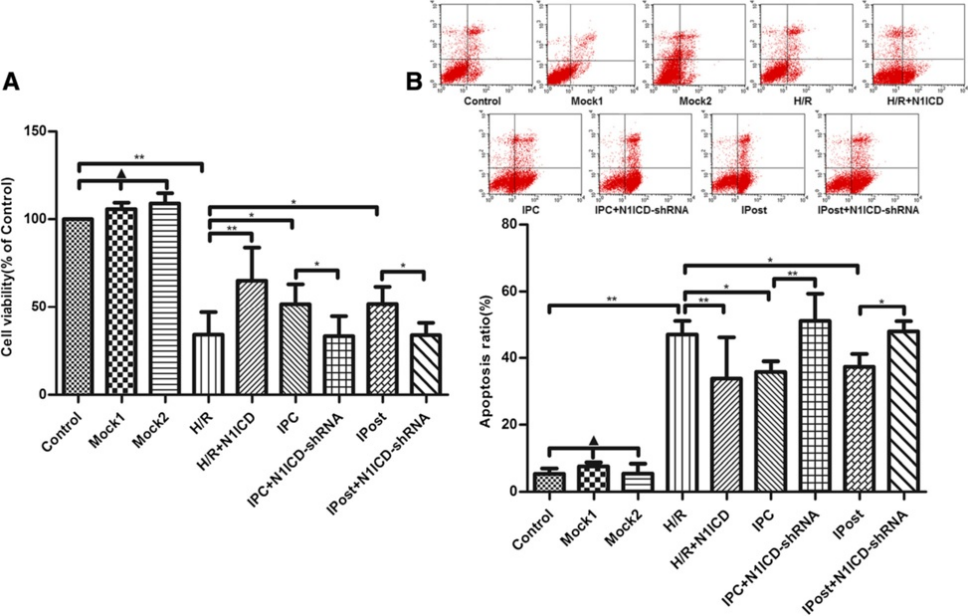
**Myocardial IRI is mitigated by activated Notch1 signaling. A**, Myocardial cell viability was enhanced by ectopic expression of N1ICD and inhibited by knockdown of N1ICD. **B**, IRI induced apoptosis was inhibited by ectopic expression of N1ICD but promoted by knockdown of N1ICD. The apoptosis of H9c2 cells was detected based on Annexin V/FITC and PI staining and flow cytometry. Data were presented as mean±SD, n=3. Mock1 indicated cells infected with empty vector, Mock2 indicated cells infected with vector containing nonspecfic sequences. ▲*P*>0.05, *P<0.05, **P<0.01.

To investigate the role of Notch1 signaling in myocardial protection, the cell viability was detected after H9c2 cells were exposed to H/R, IPC or IPost. Ectopic expression of N1ICD in H9c2 cells exposed to H/R enhanced myocardial cell viability, however, the viability of myocardial cells exposed to IPC and IPost was decreased when N1ICD was knockdown by LV-N1ICD-shRNA (Figure 3A).

Next, we observed the effect of Notch1 signaling on cell apoptosis after myocardial ischemia. The results demonstrated that the apoptosis of cardiomyocyte in H/R was greatly reduced after N1ICD overexpression, but it was substantially increased upon N1ICD knockdown during IPC and IPost (Figure 3B). Taken together, these data suggest that Notch1 signaling mitigates myocardial IRI by promoting the viability and inhibiting the apoptosis of cardiomyocytes.

### Notch1 signaling inhibits the mitochondrial permeability transition pore (mPTP) opening in cardiomyocytes

**Figure 4:**
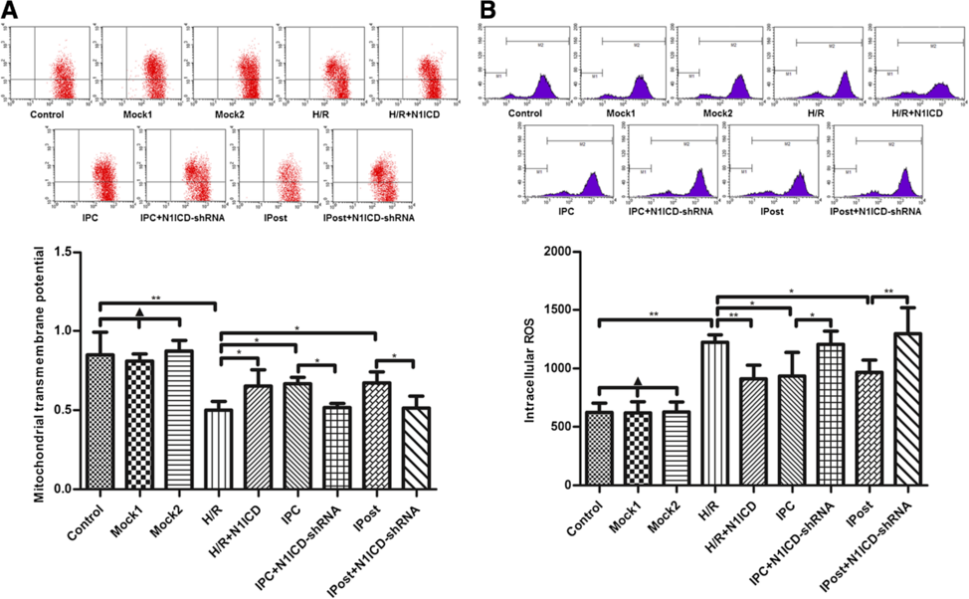
**Notch1 signaling inhibits mitochondrial mPTP opening in H9c2 cells exposed to ischemia. A**, Measurement of ΔΨm in different groups of H9c2 cells. **B**, Measurement of ROS accumulation in different groups of H9c2 cells. Data were presented as mean±SD, n=3. Mock1 indicated cells infected with empty vector, Mock2 indicated cells infected with vector containing nonspecfic sequences. ▲*P*>0.05, *P<0.05, **P<0.01.

To observe the effect of Notch1 on mPTP during IRI, we detected ΔΨm by JC-1 staining. We observed that LV-N1ICD infection during H/R prevented mitochondrial from serious depolarization, while LV-N1ICD-shRNA infection during IPC and IPost exerted opposite effect, ΔΨm became extremely low that showed no significant difference compared with H/R (Figure 4A). These data indicate that activated Notch1 suppresses the opening of mPTP.

Next we measured intracellular ROS in H9c2 cells and found that ectopic expression of N1ICD mitigated ROS accumulation caused by H/R, but knockdown of N1ICD promoted ROS accumulation during IPC and IPost (Figure 4B). These data suggest that Notch1 signaling inhibits mPTP opening in cardiomyocytes via the reduction of intracellular ROS accumulation.

### Notch1 signaling activates Stat3 and regulates apoptosis related proteins in cardiomyocytes

**Figure 5:**
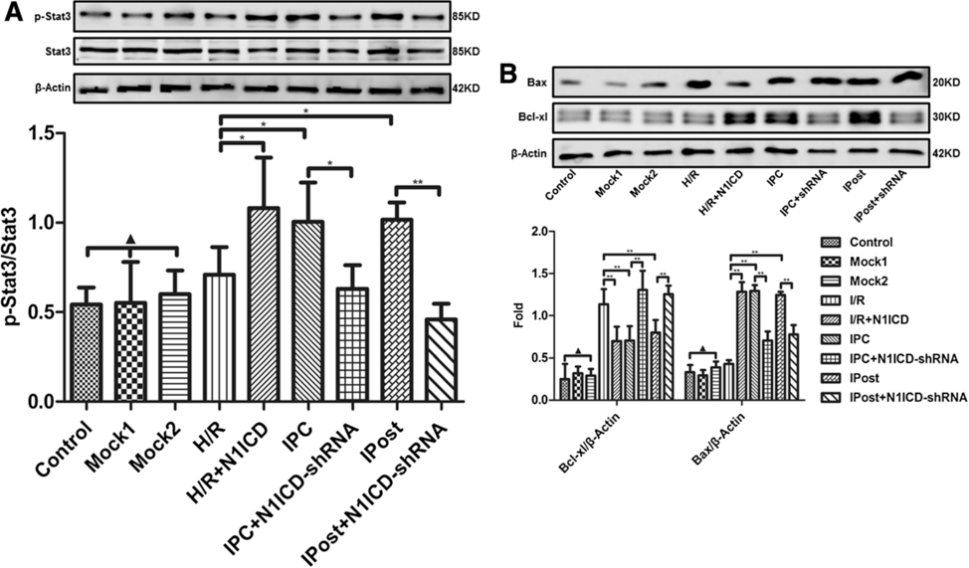
**Notch1 signaling regulates Stat3 phosphorylation and the expression of Bcl-xl and Bax in H9c2 cells exposed to ischemia. A**, The phosphorylation of Stat3 in different groups of H9c2 cells. **B**, The expression of Bcl-xl and Bax in different groups of H9c2 cells. β-actin was loading control. Data were presented as mean±SD, n=3. Mock1 indicated cells infected with empty vector, Mock2 indicated cells infected with vector containing nonspecfic sequences. ▲*P*>0.05, *P<0.05, **P<0.01.

To explore the molecular mechanism underlying Notch1 mediated cardioprotection, we detected Stat3 phosphorylation in H9c2 cells. The phosphorylation of Stat3 was elevated not only upon IPC or IPC treatment, but also in N1ICD overexpressed H9c2 cells under H/R. However, the elevated phosphorylation of Stat3 was immediately reversed upon N1ICD knockdown during IPC or IPost (Figure 5A).

Furthermore, we detected anti-apoptosis protein Bcl-xl and pro-apoptosis protein Bax in H9c2 cells. The results showed that ectopic expression of N1ICD increased Bcl-xl expression and decreased Bax expression under H/R, but N1ICD knockdown during IPC or IPost decreased Bcl-xl level and increased Bax level (Figure 5B). Collectively, these data provide evidence that cardioprotection effects of Notch1 signaling are mediated by Stat3 activation and regulation of apoptosis related proteins.

### Notch1 signaling contributes to the restoration of cardiac function in post-ischemia rats

**Figure 6:**
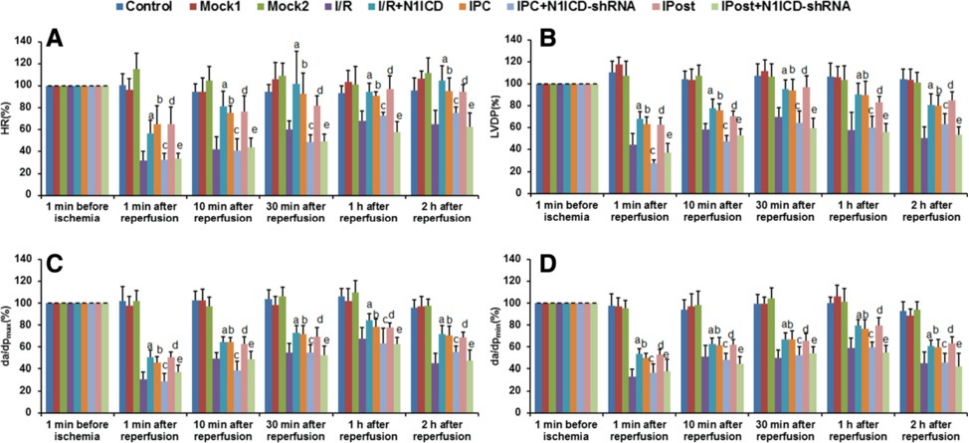
**Notch1 signaling improves cardiac function in isolated rat hearts. A-D**, Measurement of heart rate (HR), left ventricular developed pressure (LVDP) and maximal (dP/dt_max_) and minimal (dP/dt_min_) value of the first derivative of left ventricular pressure. Data were shown as the percentage of the values recorded at 1 min before ischemia. Data were presented as mean±SD, n=6. a: I/R+N1ICD vs I/R; *P*<0.05; b: IPC vs I/R, *P*<0.05; c: IPC vs IPC+shRNA, *P*<0.05; d: IPost vs I/R, *P*<0.05; e: IPost vs IPost+shRNA, *P*<0.05.

**Figure 7:**
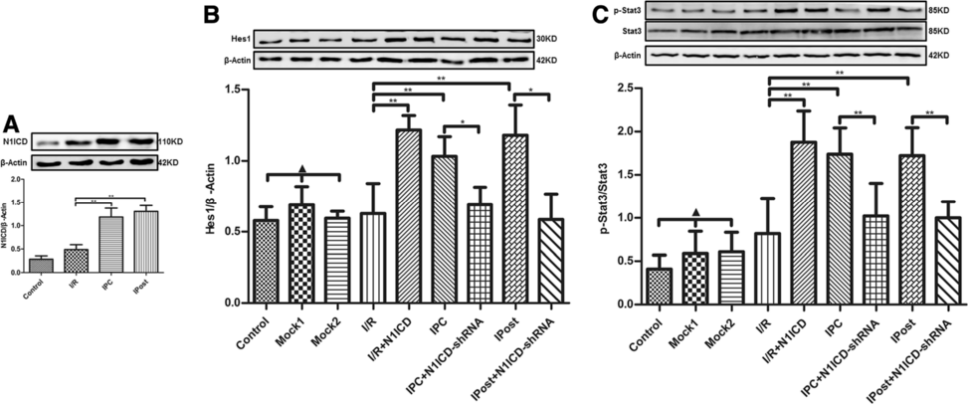
**The activation of Notch signaling in langendorff-perfused isolated heart. A**, N1ICD level was upregulated in IPC and IPost. **B**, Hes1 level was regulated by I/R, IPC and IPost with or without LV-N1ICD/LV-N1ICD-shRNA infection. **C**, The phosphorylation of Stat3 in different groups of myocardium. Data were presented as mean ± SD, n=3. Mock1 indicated the hearts infected with empty vector, Mock2 indicated the hearts infected with vector containing nonspecfic sequences. ▲*P*>0.05, *P<0.05, **P<0.01.

After langendorff-perfused isolated heart models were established, we measured the parameters of cardiac function. The results showed that the recovery of cardiac function in I/R was strengthened by ectopic expression of N1ICD, as evidenced by the increases of HR, LVDP and ±dp/dt. However, upon N1ICD knockdown, the recovery effects of IPC or IPost on cardiac function disappeared because the values of HR, LVDP and ±dp/dt were decreased to basic level of I/R (Figure 6). In addition, we found that N1ICD and Hes1 expression in myocardium was higher in IPC and Ipost, and Hes1 level was affected by LV-N1ICD or LV-N1ICD-shRNA (Figure 7A and 7B). These data suggest that Notch1 signaling is activated during IPC and IPost in langendorff-perfused isolated heart models, and Notch1 signaling contributes to the restoration of cardiac function after ischemia.

### Notch1 signaling reduces myocardial necrosis after ischemia in rats

**Figure 8:**
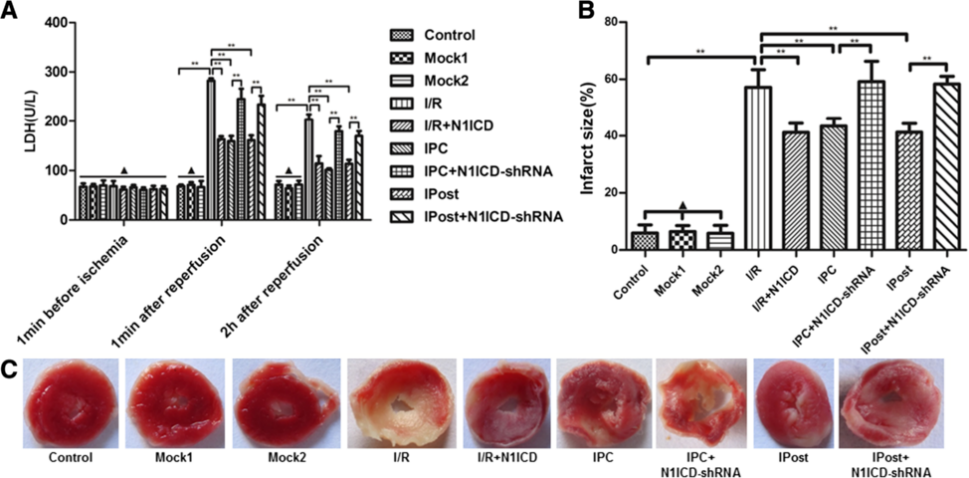
**Notch1 signaling inhibits LDH release and reduces infarct size in isolated rat hearts. A**, LDH release in each group. **B**, infarct size in each group. **C**, TTC staining of cardiac slices in each group. Data were presented as mean±SD, n=6. Mock1 indicated the hearts infected with empty vector, Mock2 indicated the hearts infected with vector containing nonspecfic sequences. ▲*P*>0.05, *P<0.05, **P<0.01.

Global ischemia for 30 min followed by 120 min of reperfusion markedly increased the release of LDH. Three episodes of IPC and IPost significantly reduced I/R induced increase in the release of LDH. Moreover, IPC caused decrease in the release of LDH was significantly restored in LV-N1ICD-shRNA infected rat hearts. However, ectopic expression of N1ICD significantly attenuated the increase in the release of LDH (Figure 8A). Similarly, the increased infarct size induced by I/R was limited by ectopic expression of N1ICD and by IPC or IPost treatment. However, N1ICD knockdown led to significant increase in infarct size during IPC and IPost (Figure 8B and 8C). These data indicate that Notch1 signaling can reduce myocardial necrosis after ischemia. Furthermore, the phosphorylation of Stat3 was enhanced by IPC and IPost treatment, or by N1ICD overexpressed in I/R. However, the elevated phosphorylation of Stat3 was obviously decreased upon N1ICD knockdown during IPC or IPost (Figure 7C).

## Discussion

The activation of Notch signaling following tissue injury has been documented in various tissues [14,15]. In this study we observed that the expression of N1ICD and Hes1 was greatly increased in H9c2 cells exposed to IPC and IPost, but not to H/R, suggesting that endogenous cardioprotection phenomenon is associated with the activation of Notch signaling. These data are consistent with recent findings that Notch1 signaling is implicated in myocardial protection mediated by IPC [16]. In addition, we found that IR-induced necrosis and apoptosis in H9c2 cardiomyocytes were mitigated by activated Notch1 signaling. Furthermore, in langendorff isolated heart perfusion model, we demonstrated that activation of Notch1 not only reduced the release of LDH and limited the infarct size, but also improved cardiac function after ischemia. Collectively, these data suggest that Notch1 acts as an endogenous cardioprotcetive factor to play an important role in the protection of ischemic myocardium during IPC and IPost.

mPTP is a non-specific channel in the mitochondrial inner membrane. Experimental evidences suggest that irreversible opening of mPTP is a critical mediator of lethal IRI, but IPC and IPost can direct or indirectly inhibit the opening of mPTP by various survival signaling pathway [17]. In the present study, we monitored the mPTP opening by ΔΨm and found that activated Notch1 alleviated the loss of ΔΨm induced by H/R, while knockdown of N1ICD during IPC and IPost exacerbated the loss of ΔΨm. These results indicate that the mPTP opening induced by H/R is partly inhibited by Notch1 signaling. Furthermore, we found that Notch1 decreased the production of intracellular ROS during H/R, in agreement with previous results that Notch1 signaling protected hepatocytes from IRI by repressing ROS [18].

Cardiomyocyte apoptosis is a complex molecular event controlled by a variety of apoptosis related proteins such as Bcl-2 family. The Bcl-2 family consists of pro-apoptotic factors such as Bad, Bid and Bax, and anti-apoptotic factors such as Bcl-2 and Bcl-xl. Bax can directly induce mPTP opening, leading to the release of cytochrome C and the activation of apoptotic signaling pathways [19]. Notch signaling could upregulate Bcl-2 and Bcl-xl expression and downregulate Bax and Bim expression, thereby inhibiting apoptosis [20]. In current study, the upregulation of Bax and downregulation of Bcl-xl induced by H/R were reversed by activated Notch1 signaling, but were promoted upon knockdown of N1ICD during IPC and IPost. Therefore, we propose that Notch1 inhibits myocardial apoptosis induced by IRI through regulating the expression of Bcl-2 family members.

Several studies have shown that the activation of Stat3 in IR not only reduces cardiomyocyte death, but also attenuates adverse cardiac remodeling [21,22]. Furthermore, myocardial IPC and IPost can enhance Stat3 phosphorylation and activity, which contribute to the cardioprotection provided by IPC and IPost [23,24]. In the current study, Stat3 phosphorylation was upregulated during endogenous cardioprotection mediated by IPC and IPost, but was inhibited upon the knockdown of N1ICD. These data suggest that Notch1 signaling prevents myocardium from IRI by enhancing Stat3 phosphorylation and activation.

## Conclusions

In summary, in this study we provide a series of evidence that Notch1 signaling is activated and mediates cardioprotection provided by IPC and IPost. Notch1 signaling exerts endogenous cardioprotection to mitigate IRI through the inhibition of mPTP opening, the regulation of Bcl-2 family member expression, and the activation of Stat3. Our results suggest that Notch1 signaling may represent a potential new pharmacologic mimic for cardioprotection of IHD.

## Competing interests

The authors declare that they have no competing interests.

## Authors’ contributions

Conceived and designed the experiments: J-cL. Performed the experiments: X-lZ, LW, Q-rX. Analyzed the data: YZ. Wrote the paper: X-lZ and J-cL. All authors read and approved the final manuscript.

## References

[B1] Minamino T Cardioprotection from ischemia/reperfusion injury: basic and translational research Circ J 2012 76 1074 1082 10.1253/circj.CJ-12-0132 22504127

[B2] Gerczuk PZ Kloner RA An update on cardioprotection: a review of the latest adjunctive therapies to limit myocardial infarction size in clinical trials J Am Coll Cardiol 2012 59 969 978 10.1016/j.jacc.2011.07.054 22402067

[B3] Apostolakis E Baikoussis NG Papakonstantinou NA The role of myocardial ischaemic preconditioning during beating heart surgery: biological aspect and clinical outcome Interact Cardiovasc Thorac Surg 2012 14 68 71 10.1093/icvts/ivr024 22108934 PMC3420290

[B4] Zhao ZQ Corvera JS Halkos ME Kerendi F Wang NP Guyton RA Vinten-Johansen J Inhibition of myocardial injury by ischemic postconditioning during reperfusion: comparison with ischemic preconditioning Am J Physiol Heart Circ Physiol 2003 285 H579 588 12860564 10.1152/ajpheart.01064.2002

[B5] Huang C Yitzhaki S Perry CN Liu W Giricz Z Mentzer RM Jr Gottlieb RA Autophagy induced by ischemic preconditioning is essential for cardioprotection J Cardiovasc Transl Res 2010 3 365 373 10.1007/s12265-010-9189-3 20559777 PMC2899015

[B6] Babiker FA van Golde J Vanagt WY Prinzen FW Pacing postconditioning: impact of pacing algorithm, gender, and diabetes on its myocardial protective effects J Cardiovasc Transl Res 2012 5 727 734 10.1007/s12265-012-9390-7 22826102

[B7] Schwanbeck R Martini S Bernoth K Just U The notch signaling pathway: molecular basis of cell context dependency Eur J Cell Biol 2011 90 572 581 10.1016/j.ejcb.2010.10.004 21126799

[B8] Kopan R Notch signaling Cold Spring Harb Perspect Biol 2012 4 10 doi:10.1101/cshperspect.a011213 10.1101/cshperspect.a011213 PMC3475170 23028119

[B9] Gude NA Emmanuel G Wu W Cottage CT Fischer K Quijada P Muraski JA Alvarez R Rubio M Schaefer E Sussman MA Activation of notch-mediated protective signaling in the myocardium Circ Res 2008 102 1025 1035 10.1161/CIRCRESAHA.107.164749 18369158 PMC3760732

[B10] Kratsios P Catela C Salimova E Huth M Berno V Rosenthal N Mourkioti F Distinct roles for cell-autonomous notch signaling in cardiomyocytes of the embryonic and adult heart Circ Res 2010 106 559 572 10.1161/CIRCRESAHA.109.203034 20007915

[B11] Croquelois A Domenighetti AA Nemir M Lepore M Rosenblatt-Velin N Radtke F Pedrazzini T Control of the adaptive response of the heart to stress via the notch1 receptor pathway J Exp Med 2008 205 3173 3185 10.1084/jem.20081427 19064701 PMC2605223

[B12] Collesi C Zentilin L Sinagra G Giacca M Notch1 signaling stimulates proliferation of immature cardiomyocytes J Cell Biol 2008 183 117 128 10.1083/jcb.200806091 18824567 PMC2557047

[B13] Campa VM Gutierrez-Lanza R Cerignoli F Diaz-Trelles R Nelson B Tsuji T Barcova M Jiang W Mercola M Notch activates cell cycle reentry and progression in quiescent cardiomyocytes J Cell Biol 2008 183 129 141 10.1083/jcb.200806104 18838555 PMC2557048

[B14] Lovschall H Tummers M Thesleff I Fuchtbauer EM Poulsen K Activation of the notch signaling pathway in response to pulp capping of rat molars Eur J Oral Sci 2005 113 312 317 10.1111/j.1600-0722.2005.00221.x 16048523

[B15] Givogri MI de Planell M Galbiati F Superchi D Gritti A Vescovi A de Vellis J Bongarzone ER Notch signaling in astrocytes and neuroblasts of the adult subventricular zone in health and after cortical injury Dev Neurosci 2006 28 81 91 10.1159/000090755 16508306

[B16] Yang Y Duan W Jin Z Bi S Yan J Jin Y Lu J Yang J Yi D New role of notch-mediated signaling pathway in myocardial ischemic preconditioning Med Hypotheses 2011 76 427 428 10.1016/j.mehy.2010.11.011 21146322

[B17] Hausenloy DJ Ong SB Yellon DM The mitochondrial permeability transition pore as a target for preconditioning and postconditioning Basic Res Cardiol 2009 104 189 202 10.1007/s00395-009-0010-x 19242644

[B18] Yu HC Qin HY He F Wang L Fu W Liu D Guo FC Liang L Dou KF Han H Canonical notch pathway protects hepatocytes from ischemia/reperfusion injury in mice by repressing reactive oxygen species production through jak2/stat3 signaling Hepatology 2011 54 979 98826 10.1002/hep.24469 21633967

[B19] Kinnally KW Antonsson B A tale of two mitochondrial channels, mac and ptp, in apoptosis Apoptosis 2007 12 857 868 10.1007/s10495-007-0722-z 17294079

[B20] Gao F Yao M Shi Y Hao J Ren Y Liu Q Wang X Duan H Notch pathway is involved in high glucose-induced apoptosis in podocytes via bcl-2 and p53 pathways J Cell Biochem 2013 114 1029 1038 10.1002/jcb.24442 23129176

[B21] Boengler K Buechert A Heinen Y Roeskes C Hilfiker-Kleiner D Heusch G Schulz R Cardioprotection by ischemic postconditioning is lost in aged and stat3-deficient mice Circ Res 2008 102 131 135 10.1161/CIRCRESAHA.107.164699 17967780

[B22] Obana M Maeda M Takeda K Hayama A Mohri T Yamashita T Nakaoka Y Komuro I Matsumiya G Azuma J Fujio Y Therapeutic activation of signal transducer and activator of transcription 3 by interleukin-11 ameliorates cardiac fibrosis after myocardial infarction Circulation 2010 121 684 691 10.1161/CIRCULATIONAHA.109.893677 20100971

[B23] You L Li L Xu Q Ren J Zhang F Postconditioning reduces infarct size and cardiac myocyte apoptosis via the opioid receptor and jak-stat signaling pathway Mol Biol Rep 2011 38 437 443 10.1007/s11033-010-0126-y 20349141

[B24] Hausenloy DJ Lecour S Yellon DM Reperfusion injury salvage kinase and survivor activating factor enhancement prosurvival signaling pathways in ischemic postconditioning: Two sides of the same coin Antioxid Redox Signal 2011 14 893 907 10.1089/ars.2010.3360 20615076

